# Vic9 mycobacteriophage: the first subcluster B2 phage isolated in Russia

**DOI:** 10.3389/fmicb.2024.1513081

**Published:** 2025-01-14

**Authors:** Marina Zaychikova, Maja Malakhova, Dmitry Bespiatykh, Maria Kornienko, Ksenia Klimina, Aleksandra Strokach, Roman Gorodnichev, Arina German, Mikhail Fursov, Dmitry Bagrov, Anna Vnukova, Alexandra Gracheva, Anastasia Kazyulina, Margarita Shleeva, Egor Shitikov

**Affiliations:** ^1^Lopukhin Federal Research and Clinical Center of Physical-Chemical Medicine of Federal Medical Biological Agency Medicine, Moscow, Russia; ^2^Federal Research Centre 'Fundamentals of Biotechnology' of the Russian Academy of Sciences, Moscow, Russia; ^3^State Research Center for Applied Microbiology and Biotechnology, Obolensk, Russia; ^4^Faculty of Biology, Lomonosov Moscow State University, Moscow, Russia; ^5^Federal State Budgetary Institution “National Medical Research Center of Phtisiopulmonology and Infectious Diseases” of the Ministry of Health of the Russian Federation, Moscow, Russia

**Keywords:** mycobacteriophages, *Mycobacterium tuberculosis*, host range, B cluster, one-step growth curve

## Abstract

Mycobacteriophages are viruses that specifically infect bacteria of the Mycobacterium genus. A substantial collection of mycobacteriophages has been isolated and characterized, offering valuable insights into their diversity and evolution. This collection also holds significant potential for therapeutic applications, particularly as an alternative to antibiotics in combating drug-resistant bacterial strains. In this study, we report the isolation and characterization of a new mycobacteriophage, Vic9, using *Mycobacterium smegmatis* mc (2)155 as the host strain. Vic9 has been classified within the B2 subcluster of the B cluster. Morphological analysis revealed that Vic9 has a structure typical of siphophages from this subcluster and forms characteristic plaques. The phage adsorbs onto host strain cells within 30 min, and according to one-step growth experiments, its latent period lasts about 90 min, followed by a growth period of 150 min, with an average yield of approximately 68 phage particles per infected cell. In host range experiments, Vic9 efficiently lysed the host strain and also exhibited the ability to lyse *M. tuberculosis* H37Rv, albeit with a low efficiency of plating (EOP ≈ 2 × 10^−5^), a typical feature of B2 phages. No lysis was observed in other tested mycobacterial species. The genome of Vic9 comprises 67,543 bp of double-stranded DNA and encodes 89 open reading frames. Our analysis revealed unique features in Vic9, despite its close relationship to other B2 subcluster phages, highlighting its distinct characteristics even among closely related phages. Particularly noteworthy was the discovery of a distinct 435 bp sequence within the gene cluster responsible for queuosine biosynthesis, as well as a recombination event within the structural cassette region (Vic_0033-Vic_0035) among members of the B1, B2, and B3 subclusters. These genetic features are of interest for further research, as they may reveal new mechanisms of phage-bacteria interactions and their potential for developing novel phage therapy methods.

## 1 Introduction

The genus Mycobacterium contains more than 200 species, among which are pathogens responsible for various infectious diseases, including tuberculosis and leprosy (Armstrong et al., 2023). In the case of tuberculosis disease caused by *Mycobacterium tuberculosis* complex (MTBC) bacteria, one of the most pressing issues is the growing resistance of MTBC strains to antibiotics. This leads to increased costs, prolonged treatment durations, and poorer outcomes for patients. The likelihood of successful treatment of diseases caused by multidrug-resistant and extensively drug-resistant strains does not exceed 60 and 30%, respectively (WHO, 2020). Consequently, tuberculosis is increasingly associated with a poor prognosis, edging closer to being classified as a conditionally incurable disease (Seung et al., 2015). Despite extensive research into novel drugs, resistance within MTBC members rapidly develops, even to the most recent drugs, such as linezolid, bedaquiline, and clofazimine (Seung et al., 2015, Kadura et al., 2020). A similar situation is observed with non-tuberculous mycobacteria (NTM), which also exhibit significant resistance to standard antibiotic therapy and, more importantly, possess innate resistance to a range of drugs, significantly complicating treatment (Saxena et al., 2021). Another challenge in infections caused by NTM is diagnostics, as standardized testing protocols are lacking in some cases, requiring specialized laboratory methods (Bartlett et al., 2024). This highlights the urgent need for novel therapeutic strategies that can be used both in conjunction with antibiotics and as stand-alone alternatives. One such approach could be phage therapy.

Bacteriophages, or simply phages, are viruses that infect bacteria and cause their lysis. The main advantages of phages include their high specificity, which allows them, unlike antibiotics, to target specific bacterial pathogens without affecting the normal microflora of the human body. Phages also have the ability to self-replicate at the site of infection, can be used in combination with other antimicrobial agents, and remain effective against drug-resistant bacterial strains (Kortright et al., 2019). Additionally, certain phages can target bacteria residing within macrophages or biofilms, which are often difficult to eradicate with conventional antibiotics (Meneses et al., 2023; Yang et al., 2024). Despite these advantages, significant barriers hinder the widespread clinical application of phages. One of the main challenges is their high specificity: unlike broad-spectrum antibiotics, phage therapy requires precise identification of the pathogen and the selection of a corresponding phage. Another critical issue is the interaction of phages with the immune system, as phages can both elicit undesirable immune responses and be neutralized by pre-existing or therapy-induced antibodies, potentially reducing their therapeutic efficacy (Kortright et al., 2019; Champagne-Jorgensen et al., 2023).

Currently, thanks to programs like Phage Hunters Integrating Research and Education (PHIRE) and Science Education Alliance Phage Hunters Advancing Genomics and Evolutionary Science (SEA-PHAGES), mycobacteriophages — phages that infect Mycobacterium genus bacteria — are among the most studied groups of phages (Heller et al., 2024). To date, about 10,000 mycobacteriophages have been identified, of which over 2,000 have been sequenced. Mycobacteriophages are classified into clusters and subclusters, with phages not included in any cluster being called singletons. According to the actinobacteriophage database PhagesDB,^1^ seven singletons and 34 clusters of mycobacteriophages have been described. Mycobacteriophages within clusters can be broadly divided into lytic and temperate phages, as phages within a cluster tend to be of the same type. However, a significant portion of known mycobacteriophages is temperate, characterized by their integration into the host genome, which limits their use in treating patients with mycobacterial infections (Hatfull, 2023).

In recent years, significant progress has been made in using mycobacteriophages as therapeutic agents. Mycobacteriophages have already been used to treat opportunistic infections caused by NTM, such as *Mycobacterium abscessus*, *Mycobacterium chelonae*, *Mycobacterium avium*, and *Mycobacterium bovis* BCG (Dedrick et al., 2019, 2021, 2023; Little et al., 2022). While human trials using mycobacteriophages to treat tuberculosis have not yet been conducted, the ongoing research shows great promise for their future use. Notably, the liposomal form of lytic mycobacteriophage D29 has shown activity against *M. tuberculosis* in both *in vitro* models of human tuberculosis granulomas and animal studies. Additionally, D29 has demonstrated efficacy as a prophylactic agent in preventing tuberculosis infection in mouse models (Avdeev et al., 2023).

These instances of successful applications of mycobacteriophages highlight the significance of their research. Further progress in this field requires creating extensive and diverse collections of mycobacteriophages, characterized by their host range, to cover a wide variety of species and strains of mycobacteria. Phages in these collections must be thoroughly characterized using microbiological methods to better understand their interactions with bacteria.

In this study, a new mycobacteriophage, Vic9, belonging to subcluster B2 of cluster B, was isolated and characterized. For this phage, as well as for other members of subcluster B2, life cycle parameters were described for the first time, and host specificity was studied across numerous Mycobacterium species. Whole-genome sequencing of Vic9 demonstrated a high level of similarity with other phages from subcluster B2; however, the phage contained several unique features that emphasize its specific genomic organization.

## 2 Materials and methods

### 2.1 Bacterial strains and culture condition

In this study we used laboratory strains of *Mycobacterium smegmatis* mc(2)155 and *Mycobacterium tuberculosis* H37Rv acquired from the A.N. Bakh Institute of Biochemistry at the Russian Academy of Science collection, as well as clinical isolates of *Mycobacterium abscessus*, *Mycobacterium fortuitum*, *Mycobacterium avium*, and *Mycobacterium kansasii* from the collection of the Federal State Budgetary Institution “NMITs FPI” of the Ministry of Health of the Russian Federation.

The mycobacteria strains were cultivated in Middlebrook 7H9 broth (Himedia, India) with 0.05% Tween-80 (Sigma-Aldrich, USA) and on solid Middlebrook 7H11 agar (Himedia, India), both containing 10% Middlebrook OADC Supplement (HiMedia, India), in a humid atmosphere with 5% CO_2_ at 37°C. For *M. smegmatis* mc(2)155, Tween-80 was not used. In experiments related to phage isolation and cultivation, Middlebrook 7H9 soft agar (0.7%) was used, and CaCl_2_ was added to all media at a final concentration of 2 mM for *M. smegmatis* mc(2)155 and 1 mM for other mycobacteria. All manipulations with bacterial strains were performed under biosafety level 2 and 3 conditions.

### 2.2 Isolation of mycobacteriophage

The *M. smegmatis* mc(2)155 strain was used as a host for phage isolation. The isolation of Vic9 phage was carried out as previously described (Gong et al., 2021). Briefly, a soil sample (10 g) was co-incubated with 1 mL of *M. smegmatis* mc(2)155 suspension grown to mid-log phase and 20 μL of ampicillin (50 mg/mL) in 10 mL MP buffer (50 mM Tris–HCl; 150 mM NaCl; 10 mM MgCl_2_; 2 mM CaCl_2_; pH 7.5) overnight with agitation. After incubation, the sample was centrifuged at 5,000 g for 10 min at 4°C. The supernatant was first filtered through a 0.45 μm PES membrane (Millipore, USA), and then through a 0.22 μm PES membrane (Millipore, USA). The filtrate (10 mL) was added to 10 mL of double-concentrated TSB (Himedia, India), and inoculated with 2 mL of *M. smegmatis* mc(2)155 suspension grown to mid-log phase and incubated for 48 h with agitation. The double-layer agar plate method was used to detect and isolate the phage (Kropinski et al., 2009). The resulting lysate was serially diluted in Middlebrook 7H9 broth, then 100 μL aliquots were mixed with soft agar inoculated with *M. smegmatis* mc(2)155 grown to mid-log phase and poured onto a prewarmed Middlebrook 7H11 agar plate. Three rounds of single plaque purification and re-infection of exponentially growing host strains yielded pure phage suspensions. Subsequently, phage lysates were stored at 4°C.

### 2.3 Phage titer

The phage titer was evaluated by standard spot test methodology (Sarkis and Hatfull, 1998). Serial tenfold dilutions of the phage were spotted in 5 μL drops on double-layer agar containing 100 μL of *M. smegmatis* mc(2)155 suspension grown to mid-log phase.

### 2.4 Electron microscopy of phage

The phage lysate was filtered through a 0.22 μm PES membrane (Millipore, USA) and concentrated by ultracentrifugation at 75,000 g for 1 h at 4°C. The phage particles were resuspended in SM buffer (50 mM Tris–HCl pH 7.5, 100 mM NaCl, 8 mM MgSO_4_, 0.01% gelatin). A suspension of phages (10^12^ plaque-forming units (PFU)/mL) was deposited onto carbon-coated grids (Ted Pella, USA) treated using a glow discharge device K100X (Quorum Technologies Ltd., Lewes, UK). Following one-minute deposition, the suspension was blotted, and the grids were stained with 1% uranyl acetate and subsecuently dried. The images were acquired using a transmission electron microscope (TEM) JEM-1400 (JEOL, Japan) operating at 120 kV.

### 2.5 Optimal multiplicity of infection

Mid-log phase *M. smegmatis* mc(2)155 cells were inoculated in Middlebrook 7H9 broth to a concentration of 1 × 10^7^ CFU/mL. The phage was mixed with bacteria at multiplicities of infection (MOIs) of 0.001, 0.01, 0.1, 1, and 10. After incubation with shaking for 48 h, the phage titer was determined using the spot test method (Sarkis and Hatfull, 1998). The experiment was performed in three biological replicates.

### 2.6 Adsorption curve

The phage adsorption capacity was determined as previously described (Hendrix and Duda, 1992). Briefly, *M. smegmatis* mc(2)155 in mid-log phase and Vic9 phage were mixed at a multiplicity of infection (MOI) of 0.01 in Middlebrook 7H9 broth to a total volume of 5 mL. The mixture was incubated with agitation, and 100 μL aliquots were collected every 10 min over a 1-h period. Each aliquot was immediately centrifuged at 13,800 g for 2 min. The titer of non-adsorbed phage in the supernatant was evaluated using the double-layer agar plate method (Kropinski et al., 2009) as described previously. The experiment was carried out in three biological replicates.

### 2.7 One-step growth curve

The one-step growth curve of Vic9 phage was carried out as previously described (Gan et al., 2014), with modifications. *M. smegmatis* mc(2)155 in mid-log phase and Vic9 phage were mixed at a MOI of 0.01 in a total volume of 0.9 mL and incubated at 37°C for 30 min to allow phage adsorption. To inactivate unbound phages, 0.1 mL of 100 mM ferrous ammonium sulfate (Sigma-Aldrich, USA) was added to the mixture, following the optimal concentration previously demonstrated to be most effective for phage inactivation (McNerney et al., 1998). After incubation at room temperature for 5 min, the mixture was centrifuged at 13,800 g for 2 min. The pellet was resuspended in 1 mL of Middlebrook 7H9 broth, and the resulting suspension was used to determine the number of infected cells and to construct a one-step growth curve.

The number of infected cells was determined using the double-layer agar plate method (Kropinski et al., 2009) with modifications. Briefly, an aliquot of the suspension, diluted from 10^–1^ to 10^–7^, was added to soft agar, which additionally contained *M. smegmatis* mc(2)155 in mid-log phase for better visualization. The number of plaques formed was equal to the number of infected cells.

To construct the one-step growth curve, the resulting suspension (100 μL) was additionally diluted 100-fold in Middlebrook 7H9 broth and incubated with agitation. Aliquots were collected every 30 min for 5 h and centrifuged at 13,800 g for 2 min. The titer of free phage in the supernatant was assessed using the double-layer agar plate method (Kropinski et al., 2009). The experiment was carried out in three biological replicates.

The burst size was determined as the number of phages released from each infected cell, calculated by the ratio of the final number of released phage particles to the initial number of infected bacterial cells.

### 2.8 Host range determination

Phage activity against mycobacterial strains was determined by spot testing on double-layer agar (Sarkis and Hatfull, 1998). For this, 1 mL of the test bacterial culture suspension was washed twice with fresh Middlebrook 7H9 broth to remove Tween-80. The pellet was resuspended in 100 μL of Middlebrook 7H9 broth and added to 5 mL of soft agar and poured onto Middlebrook 7H11 agar. Tenfold serial dilutions of the phage in MP buffer (initial titer of 10^9^ PFU/mL) were spotted in 5 μL drops on the surface of the agar. The plates were incubated for 48 h for fast-growing mycobacteria and 3 weeks for slow-growing ones. The efficiency of plating (EOP) was calculated as the ratio of the phage titer on the test strain to that on the host strain *M. smegmatis* mc(2)155.

### 2.9 DNA extraction and whole-genome sequencing

Phage genomic DNA was extracted from the lysate using the standard phenol-chloroform extraction protocol (Green et al., 2012). The extracted DNA (100 ng) was used for library preparation using the KAPA HyperPlus Kit (Roche, Switzerland) according to the manufacturer’s protocol. The library underwent a final cleanup using KAPA HyperPure Beads (Roche, Switzerland), after which the library size distribution and quality were assessed using a high sensitivity DNA chip (Agilent Technologies). The libraries were then quantified using the Quant-iT DNA Assay Kit, High Sensitivity (Thermo Fisher Scientific). The DNA libraries underwent sequencing using the HiSeq 2,500 platform (Illumina, USA), in accordance with the manufacturer’s recommendations. For this purpose, following reagent kits were employed: HiSeq Rapid PE Cluster Kit v2, HiSeq Rapid SBS Kit v2 (200 cycles), and HiSeq Rapid PE FlowCell v2. Additionally, a 2% PhiX spike-in control was included in the process.

### 2.10 Bioinformatics analysis

#### 2.10.1 Genome assembly

Taxonomic confirmation of the sequenced reads was accomplished with Kraken2 v2.1.2 (Wood et al., 2019) and Bracken v2.8 (Lu et al., 2017). The quality assessment of short paired-end reads was performed using falco v1.2.1 (de Sena Brandine and Smith, 2019) and MultiQC v1.17 (Ewels et al., 2016). Adapters removal and reads filtering was performed using fastp v0.23.4 (Chen et al., 2018). The genome of the Vic9 phage was assembled using Unicycler v0.5.0 and SPAdes v3.15.5. Completeness quality of the assembled phage genome was assessed with CheckV v1.0.1 (Nayfach et al., 2021). Prokka v1.14.6 (Seemann, 2014) and Pharokka v1.7.3 (Bouras et al., 2023) were utilized to annotate the genome’s assembly. Subsequently, the annotation was manually curated using GeneMarkS v4.32 (Besemer et al., 2001) to identify open reading frames. ARAGORN v1.2.41 (Laslett and Canback, 2004) was employed to search for tRNA-coding genes. Further gene annotations were carried out using BLASTp v2.13.0 and HHPred (Söding et al., 2005). BWA MEM v0.7.17-r1188 (Li and Durbin, 2009) was employed for mapping reads to the assembly, subsequently SAMtools v1.17 (Danecek et al., 2021) and mosdepth v0.3.5 (Pedersen and Quinlan, 2018) were used to compile mapping statistics. The genome of the Vic 9 phage has been deposited in GenBank under accession number PP526940.2.

#### 2.10.2 Phylogenetic analysis

Mycobacteriophages genomes from PhagesDB (Russell and Hatfull, 2017) and ICTV (Lefkowitz et al., 2018) databases were retrieved using ncbi-acc-download v0.2.8 tool.^2^ Retrieved nucleotide sequences of phage genomes were aligned using Kalign 3 v3.4.0 (Lassmann, 2019) with the following parameters: “--type dna --gpo 11 --gpe 0.85 --tgpe 0.45.” The resulting alignment was subsequently trimmed using trimAl v1.4.rev15 (Capella-Gutiérrez et al., 2009) with the “-gappyout” option. SeqKit2 v2.8.2 (Shen et al., 2024) was used to remove duplicated sequences from the alignment. The maximum likelihood (ML) phylogeny was inferred from 375 sequences and 54,439 distinct patterns using IQ-TREE 2 v2.3.4 (Minh et al., 2020). Support values were determined from 10,000 ultrafast bootstrap replicates UFBoot (Hoang et al., 2018) with the “-bnni” parameter and from 10,000 replicates for Shimodaira-Hasegawa (SH) approximate likelihood ratio test with the “-altr” parameter. The best-fit model was identified by ModelFinder (Kalyaanamoorthy et al., 2017) implemented in IQ-TREE with the “-m MFP” parameter, and the model selected based on the Bayesian information criterion (BIC) was GTR + F + I + R10. The resulting phylogeny was visualized using the following R v4.3.0 (R Core Team. R: A Language and Environment for Statistical Computing. 2023) packages: tidytree v0.4.5 (Yu, 2022), phylotools v1.9-16,^3^ ape v5.7–1 (Paradis and Schliep, 2019), ggnewscale v0.5.0.9000,^4^ ggplot2 v3.5.1 (Wickham, 2016), ggtree v3.8.2 (Xu et al., 2022), ggtreeExtra v1.10.0 (Xu et al., 2021), ggstar v1.0.4,^5^ cowplot v1.1.1,^6^ ggplotify v0.1.2,^7^ gtools v3.9.4,^8^ colorspace v2.1–0,^9^ and gginnards v0.2.0.^10^

Mycobacteriophages of the B cluster were clustered using vConTACT2 v0.11.3 and visualized by Cytoscape v3.10.2 using an edge-weighted spring-embedded model.

#### 2.10.3 Comparative genomics

The genomic organization of the Vic9 genome was visualized using ggplot2 v3.5.1, scales v1.3.0,^11^ gggenes v0.5.1,^12^ gggenomes v1.0.0,^13^ ggrepel v0.9.5,^14^ ggwrap v0.0.0.9001,^15^ ggpp v0.5.8–1,^16^ cowplot v1.1.1, ggtext 0.1.2.^17^ The nucleotide sequence of Vic9 was compared with other mycobacteriophages of subcluster B2 from the NCBI database using BLAST.^18^ Clinker v0.0.25^19^ was used to visualize genomic differences and to construct a comparative map of the recombinant region.

## 3 Results

### 3.1 Isolation and phenotypic characterization of the phage

**Figure 1:**
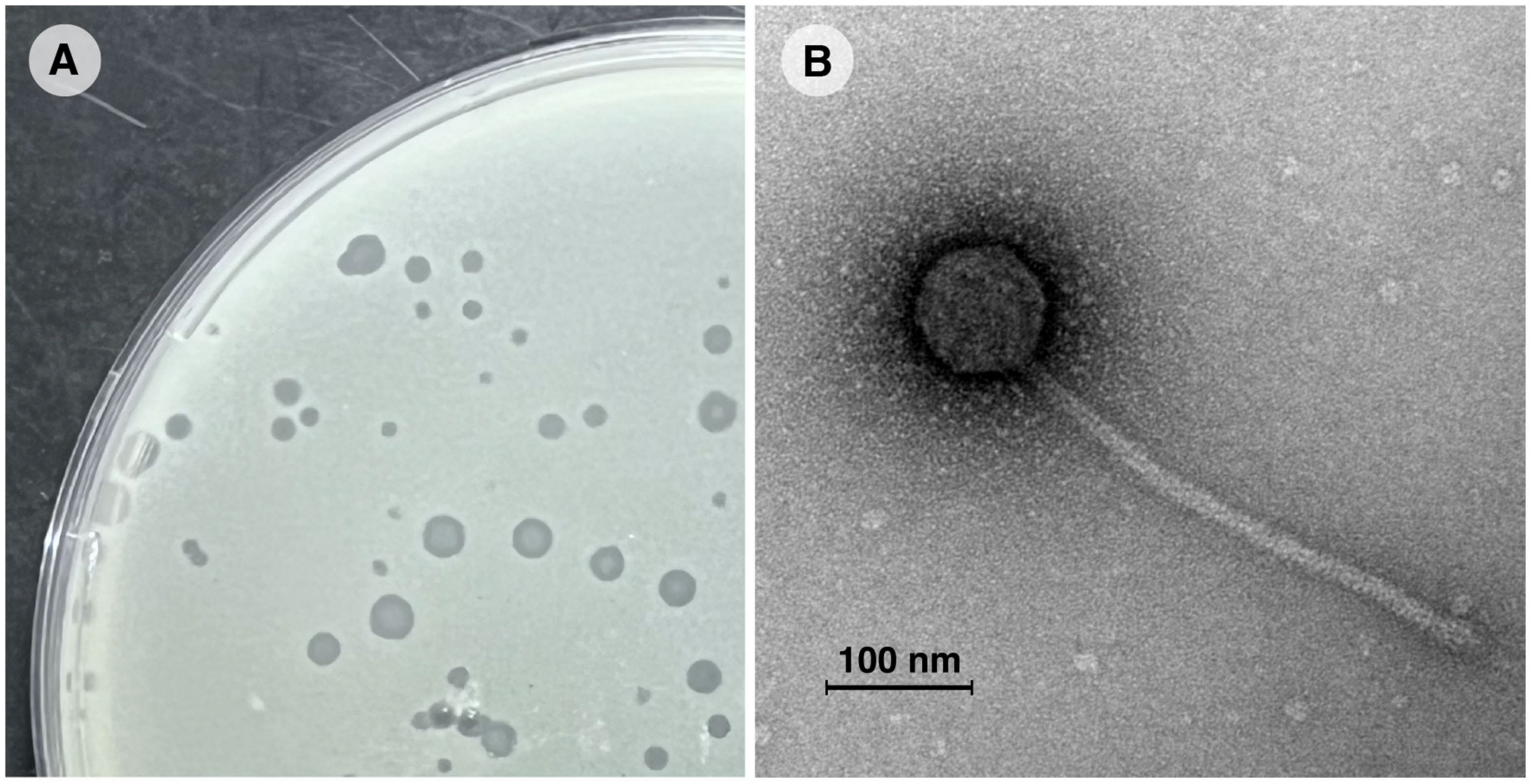
Morphological characterization of mycobacteriophage Vic9. **(A)** Plaques of phage Vic9 formed on a lawn of *M. smegmatis* mc(2)155 after 48 h of incubation. **(B)** Electron micrograph of phage Vic9, stained with 1% uranyl acetate.

Vic9 was isolated from soil samples collected at a livestock yard in the Moscow region, where small ruminants (goats) were kept. The phage plaques on the host strain *M. smegmatis* mc(2)155 were 1–3 mm in diameter, with clear edges, a round shape, and a turbid center (Figure 1A). Electron microscopy revealed that Vic9 exhibits typical siphophage morphology, characterized by an icosahedral head, 68 ± 2 nm in diameter, and a long non-contractile tail, 265 ± 10 nm in length (Figure 1B).

**Figure 2:**
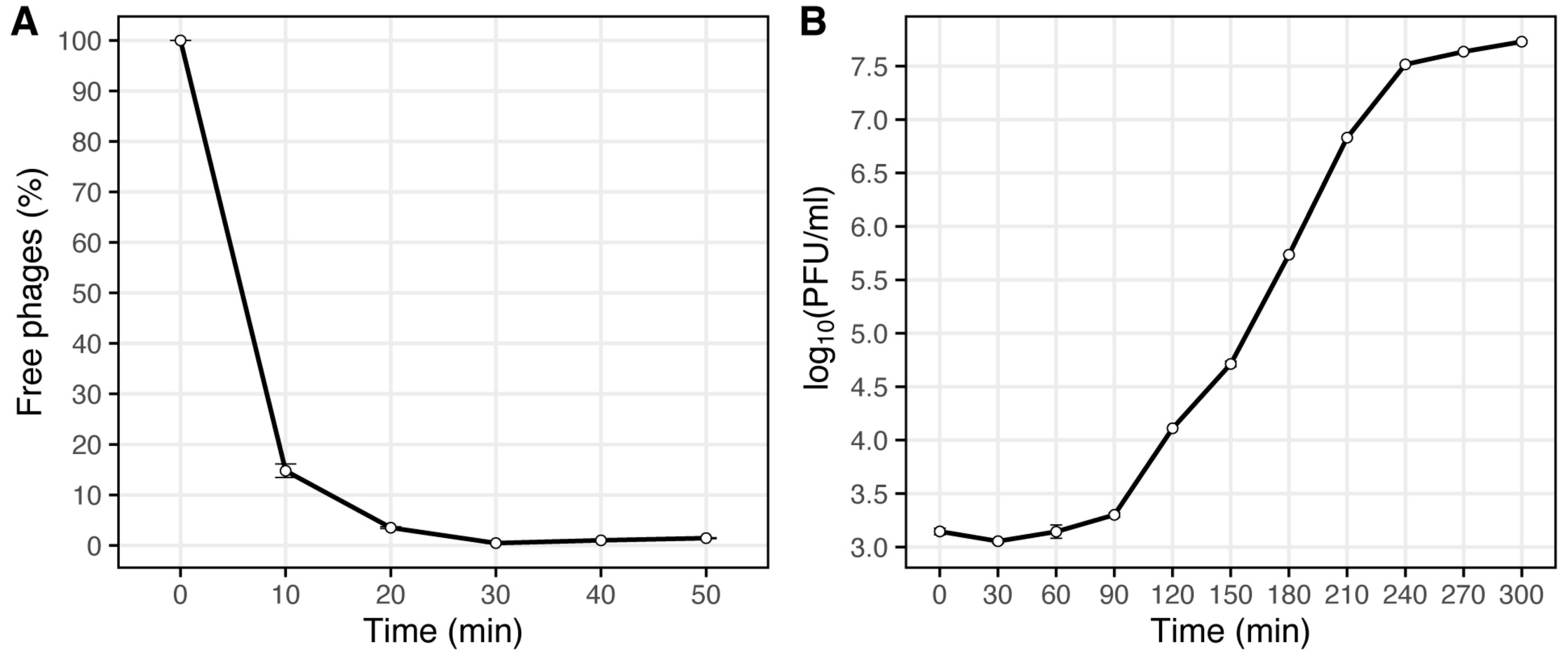
Characterization of phage Vic9 life cycle. **(A)** Adsorption curve, and **(B)** one-step growth curve.

The optimal MOI assay of the Vic9 indicated that the highest titer, 6.9 × 10^9^ PFU/mL, was achieved at a MOI of 0.01. The adsorption time of the phage to *M. smegmatis* mc(2)155 was approximately 30 min (Figure 2A). One-step growth curve analysis showed that Vic9 has a latent period of 90 min, followed by a rise period lasting 150 min (Figure 2B), with an estimated burst size of approximately 68 PFU per infected cell.

### 3.2 Host range of the Vic9 phage

**Figure 3:**
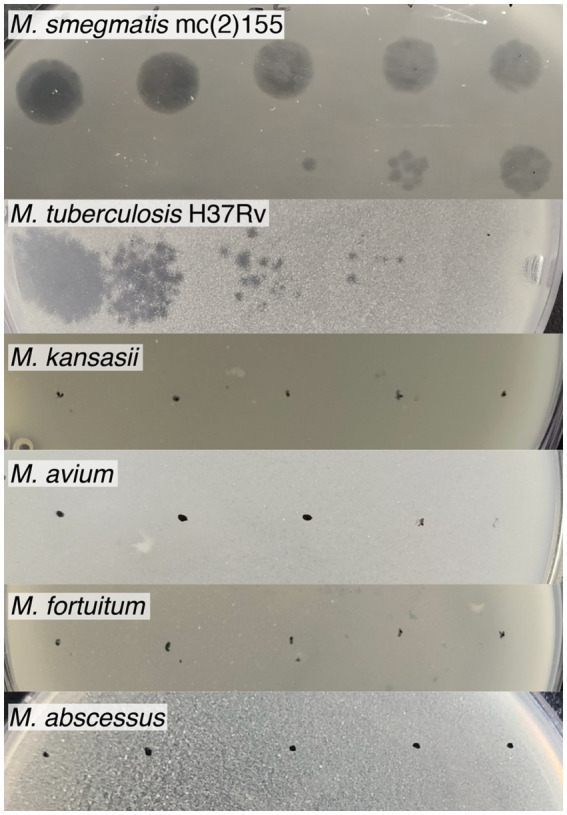
Host range of mycobacteriophage Vic9. Tenfold serial dilutions of Vic9 were spotted onto lawns of various mycobacterial species.

To determine the host range, serial dilutions of the Vic9 phage were tested on various *Mycobacterium* species (Figure 3). The phage displayed a narrow host range, effectively lysing only the host strain and *M. tuberculosis* H37Rv. However, in contrast to *M. smegmatis* mc(2)155, the phage produced small, irregularly shaped, translucent plaques with indistinct borders on *M. tuberculosis* H37Rv. No lysis was observed on *M. kansasii*, *M. avium*, *M. fortuitum*, or *M. abscessus*, regardless of the phage titer.

### 3.3 General genome analysis of the phage

The complete genome of the Vic9 phage consists of linear double-stranded DNA, 67,543 bp in length, with a G + C content of 69%. A total of 89 open reading frames (ORFs) were identified, covering 63,351 bp (94% of the genome).

**Figure 4:**
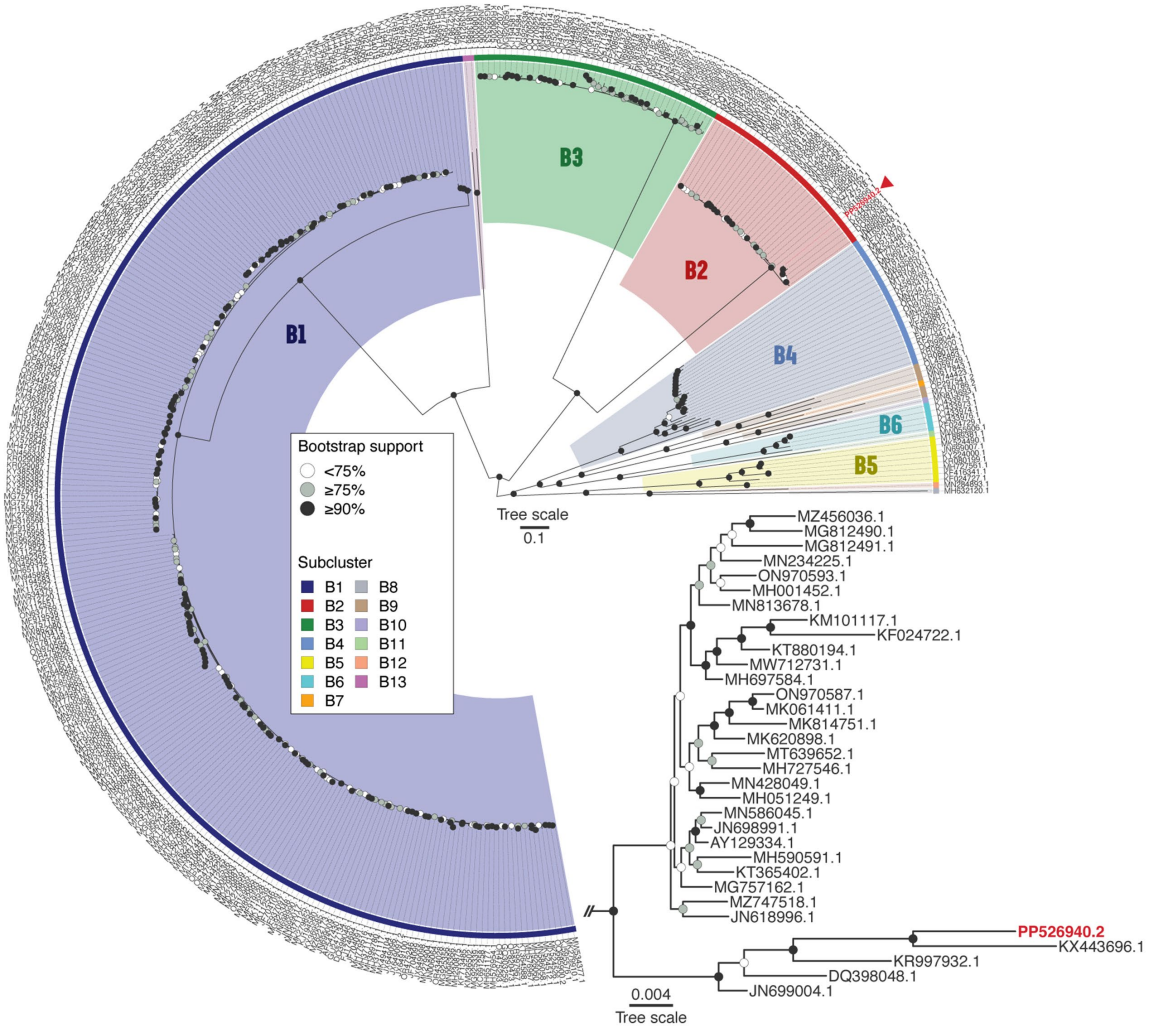
Phage Vic9 is classified within a distinct phylogenetic clade of the B2 subcluster. The midpoint-rooted maximum-likelihood phylogeny was inferred from 375 sequences and 54,439 distinct patterns obtained through whole-genome alignments of nucleotide sequences from cluster B mycobacteriophages. Bootstrap support values are indicated as white dots for values <75%, grey dots for values ≥75%, and black dots for values ≥90% on the interior nodes. Colors represent the various subclusters of cluster B, with the red triangle marking the Vic9 phage. The subtree for subcluster B2 is shown in the lower right corner.

BLASTn analysis revealed that Vic9 shares a high degree of similarity with other phages of subcluster B2, showing over 95% query coverage and 98.9% identity. To verify its taxonomic position, a phylogenetic analysis of the phage, along with phages of cluster B (*n* = 375) from the actinobacteriophage database PhagesDB (Russell and Hatfull, 2017), was conducted. Phylogenetic analysis confirmed its affiliation with subcluster B2, with Vic9 forming a distinct group with the phages Godines (KR997932.1), Ares (JN699004.1), Qyrzula (DQ398048.1), and Laurie (KX443696.1) (Figure 4).

### 3.4 Functional annotation of Vic9 phage

**Figure 5:**
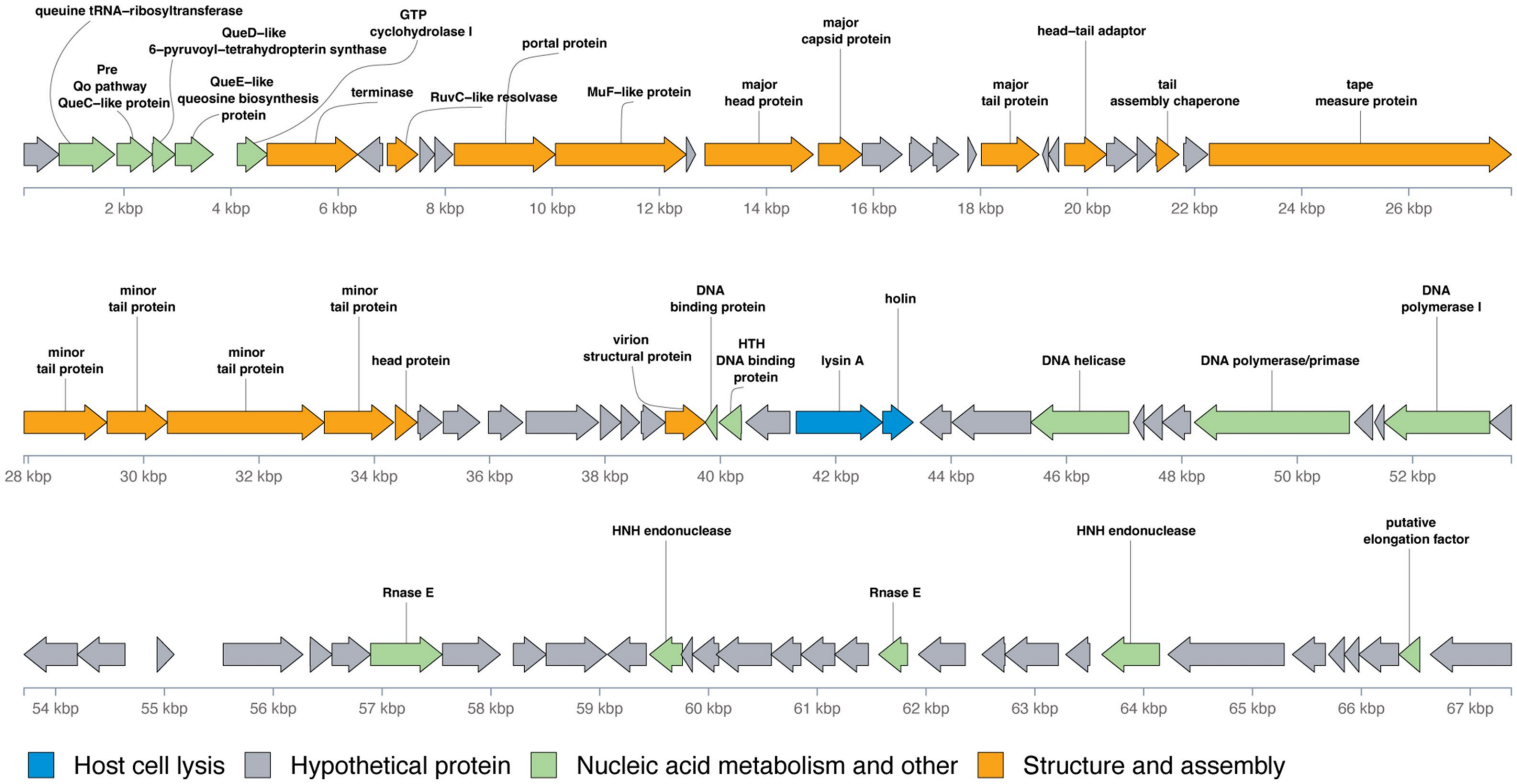
Genomic organization of mycobacteriophage Vic9. Open reading frames (ORFs) are represented as arrows, with translation frames indicated. Functional annotations of the ORFs are color-coded as follows: blue for host cell lysis, grey for hypothetical proteins, green for nucleic acid metabolism and other functions, and orange for structure and assembly.

The genomic organization of phage Vic9 is typical of subcluster B2. Functional analysis categorized its proteins into four main groups: (1) Structure and assembly (16 ORFs); (2) Host cell lysis (2 ORFs); (3) Nucleic acid metabolism and other functions (15 ORFs); and (4) Hypothetical proteins (56 ORFs) (Figure 5). The structural genes are located in the left region of the genome and are oriented on the positive strand. This module comprises several key genes, including those encoding the terminase (Vic_00007), RuvC-like resolvase (Vic_00009), portal protein (Vic_00012), MuF-like protein (Vic_00014), major head protein (Vic_00015), major capsid protein (Vic_00016), major tail protein (Vic_00021), head-tail adaptor (Vic_00024), tail assembly chaperone (Vic_00027), tape measure protein (Vic_00029), minor tail proteins (Vic_00030-Vic_00033), head protein (Vic_00034), and virion structural protein (Vic_00042). The tape measure protein, Vic_00029, plays a crucial role in determining tail length and is composed of 1,880 amino acid residues, consistent with the observed morphology of the phage (Belcaid et al., 2011). The cell lysis module, responsible for completing the lytic cycle and releasing phage particles from the host, contains only lysin A (Vic_00058) and holin (Vic_00059).

Preceding the structural module located in the left region of the genome, a group of genes involved in nucleic acid metabolism and other functions were identified, including genes for queuosine biosynthesis from GTP. These genes include queuine tRNA-ribosyltransferase (Vic_00002), Pre-Q0 pathway QueC-like protein (Vic_00003), QueD-like 6-pyruvoyl-tetrahydropterin synthase (Vic_00004), QueE-like queuosine biosynthesis protein (Vic_00005), and GTP cyclohydrolase I (Vic_00006). Additional genes related to nucleic acid metabolism are mainly located in the right region of the genome and include DNA-binding proteins (Vic_00043, Vic_00044), DNA helicase (Vic_00050), DNA polymerase/primase (Vic_00054), DNA polymerase I (Vic_00057), Rnase E (Vic_00065), HNH endonucleases (Vic_00070, Vic_00082), and a putative elongation factor (Vic_00088).

A distinct feature of B2 subcluster members, including Vic9, is the absence of integrase, RNA polymerase, lysin B, the small terminase subunit, and tRNA-coding genes (Hatfull, 2010).

### 3.5 Comparative genomics of Vic9

Comparison of the nucleotide sequence of Vic9 with other mycobacteriophages of subcluster B2 from the NCBI database revealed several distinct features (Supplementary Figure S1). Vic9 contains a unique 435 bp sequence located between the genes Vic_00005 (QueE-like queuosine biosynthesis protein) and Vic_00006 (GTP cyclohydrolase I). This sequence lacks open reading frames when annotated with GeneMarkS v4.32. However, analysis using Pharokka 1.7.2 revealed that this region encodes a protein of unknown function. Notably, no homologs of this sequence or the putative protein it encodes were identified in the NCBI database. Additionally, Vic9 possesses the gene Vic_00055, encoding a protein of unknown function, homologous to FDI79_gp56 (96.15%) from phage Godines (KR997932.1) and J3996_gp55 (98.08%) from phage Laurie (KX443696.1), but absent in other cluster members. Additionally, Vic9 is missing the region between Vic_00087 and Vic_00088, which encodes a hypothetical protein in other mycobacteriophages. Notably, this deletion is also present in the phages Laurie (KX443696.1), Arbiter (JN618996.1), Phantasmagoria (ON970587.1), Holeinone (MG812490.1), and Qyrzula (DQ398048.1).

**Figure 6:**
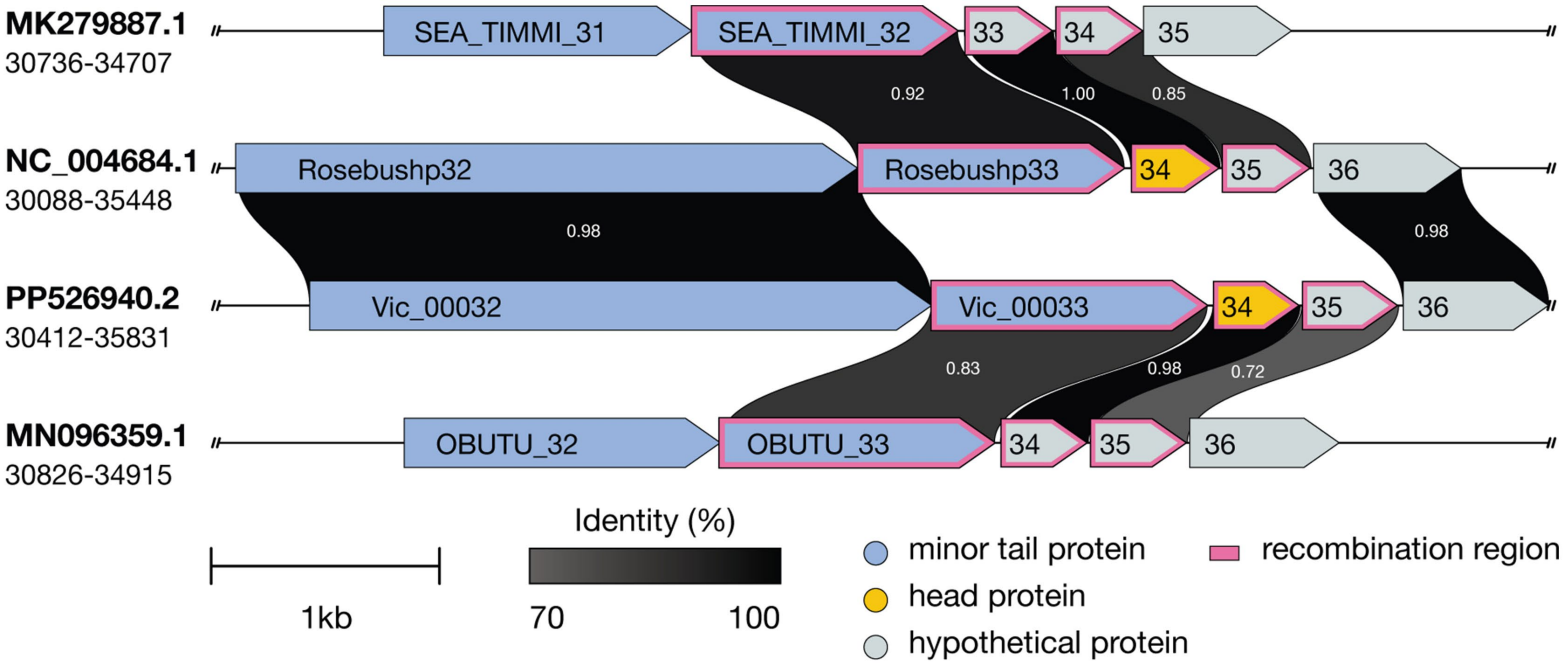
Comparative genomic organization of mycobacteriophages Timmi, Rosebush, Vic9, and Obutu reveals evidence of recombination events. Recombination events are indicated by a pink border. ORFs are represented by arrows, with grayscale shading indicating amino acid sequence identity (darker shades correspond to higher identity levels). Accession numbers and genome coordinates are noted on the left.

Further genomic analysis revealed that the structural cassette region Vic_0033-Vic_0035, comprising a minor tail protein, a head protein, and a protein of unknown function, shares homology with the corresponding regions in phages Godines (KR997932.1), Ares (JN699004.1), Qyrzula (DQ398048.1), and Laurie (KX443696.1). This homology places Vic9 on a distinct branch within subcluster B2. Additionally, Vic_0033-Vic_0035 showed significant homology with phages from subcluster B3, as indicated by the analysis of 28 out of 44 genomes from PhagesDB, which displayed over 75% similarity. Notably, no homology with other subcluster B2 members was observed (Figure 6).

## 4 Discussion

A novel lytic mycobacteriophage, Vic9, was isolated and thoroughly characterized using *M. smegmatis* mc(2)155 as the host. Vic9 belongs to subcluster B2 within the larger B cluster, which, according to the PhagesDB, is the second most represented cluster after cluster A, and the most prevalent among phages with a lytic life cycle. The B cluster comprises 13 subclusters, with subcluster B2 being the third largest, following subclusters B1 and B3 (as of October 2024). Despite the relatively large number of studied phages in subcluster B2, none have had their life cycle characteristics described, which are essential for understanding phage physiology and evaluating their therapeutic potential.

Morphologically, Vic9 is a typical representative of cluster B and, like most mycobacteriophages, belongs to the Siphoviridae family. On its host strain, Vic9 forms small plaques with a turbid center, which is a characteristic feature of B2 subcluster members, according to PhagesDB. In terms of host specificity, Vic9 efficiently lyses its host strain, *M. smegmatis* mc(2)155, and at higher titers, it also demonstrates lysis of *M. tuberculosis* H37Rv, albeit with a low efficiency of plating (EOP of 2 × 10^−5^). This observation aligns with previous studies noting the relatively low efficacy of B2 subcluster phages against *M. tuberculosis* (Jacobs-Sera et al., 2012), especially when compared to phages from clusters A2, A3, G1, K1, K2, K3, К4 and some singletons,which are considered the most promising for phage therapy (Guerrero-Bustamante et al., 2021; Yang et al., 2024).

Further investigation of Vic9’s life cycle parameters, compared to phage PDRPxv (from subcluster B1), the only characterized phage of the B cluster; (Sinha et al., 2020), revealed that Vic9 has a shorter adsorption time (30 vs. 45 min) and a shorter latent period (90 vs. 135 min). However, in terms of growth, Vic9 lags significantly behind one of the most studied mycobacteriophages, D29 (Bavda and Jain, 2020), which may partly explain its low efficiency in lysing mycobacteria other than *M. smegmatis*.

At the genomic level, Vic9 shares similar organization with other B2 subcluster members, as detailed elsewhere (Hatfull, 2012). According to ICTV, Vic9 is classified under the Rosebushvirus genus, named after the first phage isolated in this group (Pedulla et al., 2003). Overall, B2 subcluster mycobacteriophages show a high degree of genetic conservation and rarely exchange genetic material with other subclusters (Supplementary Figure S1). However, in Vic9 genome, as well as in phages Ares (JN699004.1), Laurie (KX443696.1), Qyrzula (DQ398048.1), and Godines (KR997932.1), we identified three genes that show homology to B3 subcluster phages (Figure 6), suggesting possible recombination between subclusters. Interestingly, the same genes in another group of phages forming a separate group on the phylogenetic tree align with corresponding regions in B1 subcluster phages (similarity >90%), which was previously reported for the Rosebush phage (Hatfull, 2010).

Another intriguing feature unique to Vic9 is the presence of a gene encoding a protein of hypothetical function located at the left end of the genome, within a region of six genes preceding the terminase, previously described as specific to subcluster B2 mycobacteriophages (Hatfull, 2012). This gene lies between Vic_00005 and Vic_00006, which correspond to the QueE and QueF proteins involved in the biosynthesis of queuosine from GTP. This system likely plays a role in modifying the host’s tRNA, although it is unknown whether it affects translation specificity or efficiency.

## 5 Conclusion

The isolation and characterization of Vic9, a novel mycobacteriophage from Russia, represent a significant advancement in our understanding of subcluster B2. Until now, phages from subcluster B2 have predominantly been isolated in the United States, with the exception of phage Godines (KR997932.1) from Brazil, making the isolation of Vic9 particularly interesting for evolutionary studies. Despite high conservation within the subcluster, unique genetic features were identified, such as a distinctive sequence between genes Vic_00005 and Vic_00006, as well as homology of genes Vic_0033-Vic_0035 with corresponding genes from subcluster B3. Another key aspect of our research was the investigation of Vic9 life cycle parameters, which to our knowledge has been shown for the first time for phages of the B2 subcluster. Additionally, we found that Vic9 has a narrow host range, effectively lysing only its host strain, which significantly limits its therapeutic potential. Nonetheless, the characterization of Vic9 broadens our understanding of B2 phages and highlights their relevance for evolutionary and genetic studies, while also contributing to the growing body of knowledge needed to inform future research on the potential of mycobacteriophages in biomedical applications.

## Data Availability

The data presented in the study are deposited in the GenBank repository, accession number PP526940.2.

## Footnotes

^1^https://phagesdb.org; as of October 2024.

^2^ https://github.com/kblin/ncbi-acc-download

^3^ https://CRAN.R-project.org/package=phylotools

^4^ https://CRAN.R-project.org/package=ggnewscale

^5^ https://CRAN.R-project.org/package=ggstar

^6^ https://CRAN.R-project.org/package=cowplot

^7^ https://CRAN.R-project.org/package=ggplotify

^8^ https://CRAN.R-project.org/package=gtools

^9^ https://CRAN.R-project.org/package=colorspace

^10^ https://CRAN.R-project.org/package=gginnards

^11^ https://CRAN.R-project.org/package=scales

^12^ https://github.com/wilkox/gggenes

^13^ https://github.com/thackl/gggenomes

^14^ https://CRAN.R-project.org/package=ggrepel

^15^ https://github.com/wilkox/ggwrap

^16^ https://CRAN.R-project.org/package=ggpp

^17^ https://CRAN.R-project.org/package=ggtext

^18^ https://blast.ncbi.nlm.nih.gov/Blast.cgi

^19^ https://github.com/gamcil/clinker
