# Supplementary material for: Vic9 mycobacteriophage: the first subcluster B2 phage isolated in Russia
Source: Front Microbiol. 2025 Jan 14;15:1513081. doi: 10.3389/fmicb.2024.1513081 (PMC11772480; doi:10.3389/fmicb.2024.1513081)
Supplement: Supplementary file 1 [file Data_Sheet_1.pdf]

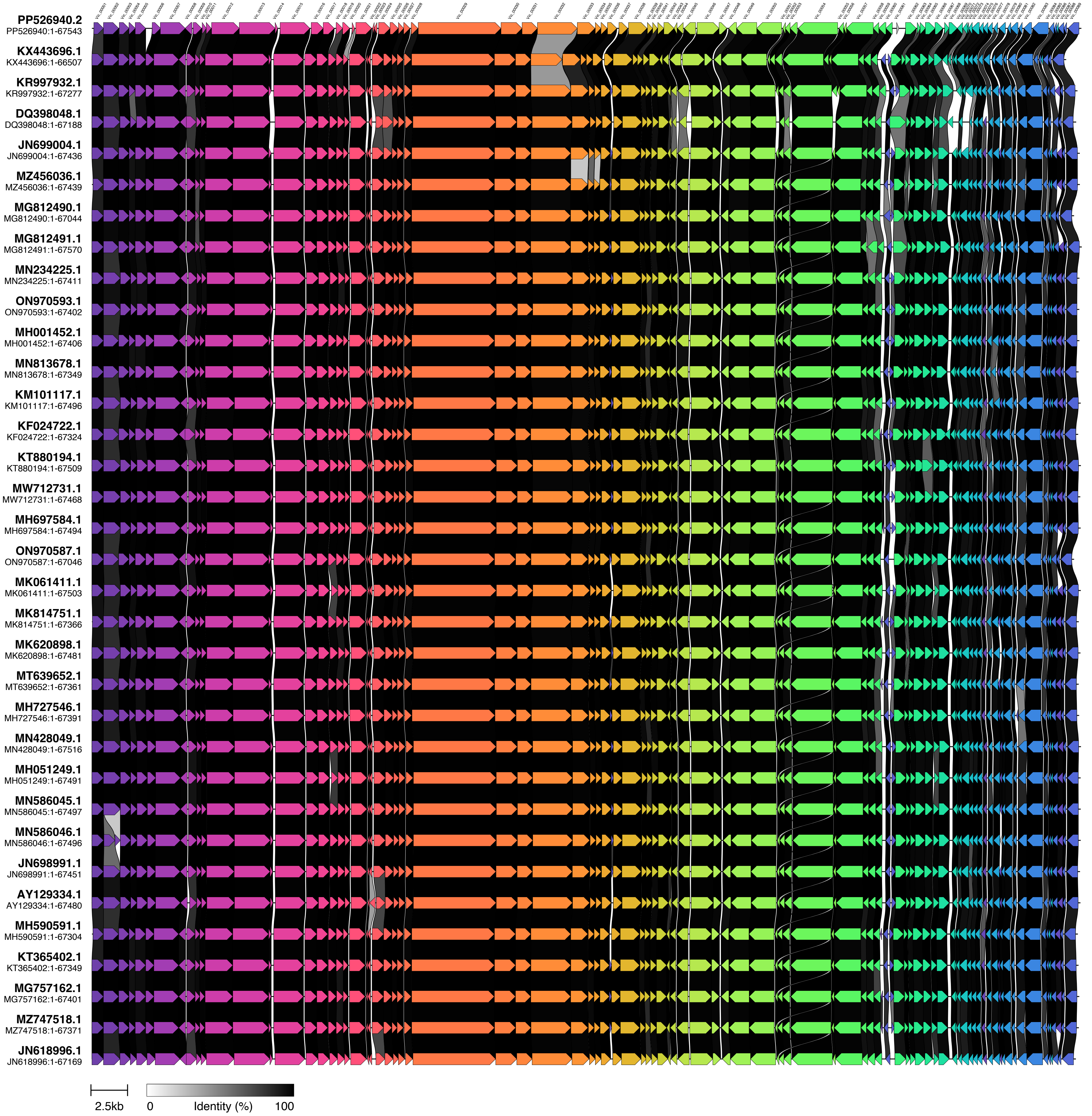

Supplementary Figure 1. Comparison of genome organization in subcluster B2. Vic 9 locus tags are shown above the genome. ORFs are represented by arrows, with shading in grayscale to indicate amino acid sequence identity (intensity corresponds to identity level). Accession numbers and genome lengths are noted on the left.
