# Supplementary material for: Vic9 mycobacteriophage: the first subcluster B2 phage isolated in Russia
Source: Front Microbiol. 2025 Jan 14;15:1513081. doi: 10.3389/fmicb.2024.1513081 (PMC11772480; doi:10.3389/fmicb.2024.1513081)
Supplement: Supplementary file 2 [file Data_Sheet_2.pdf]

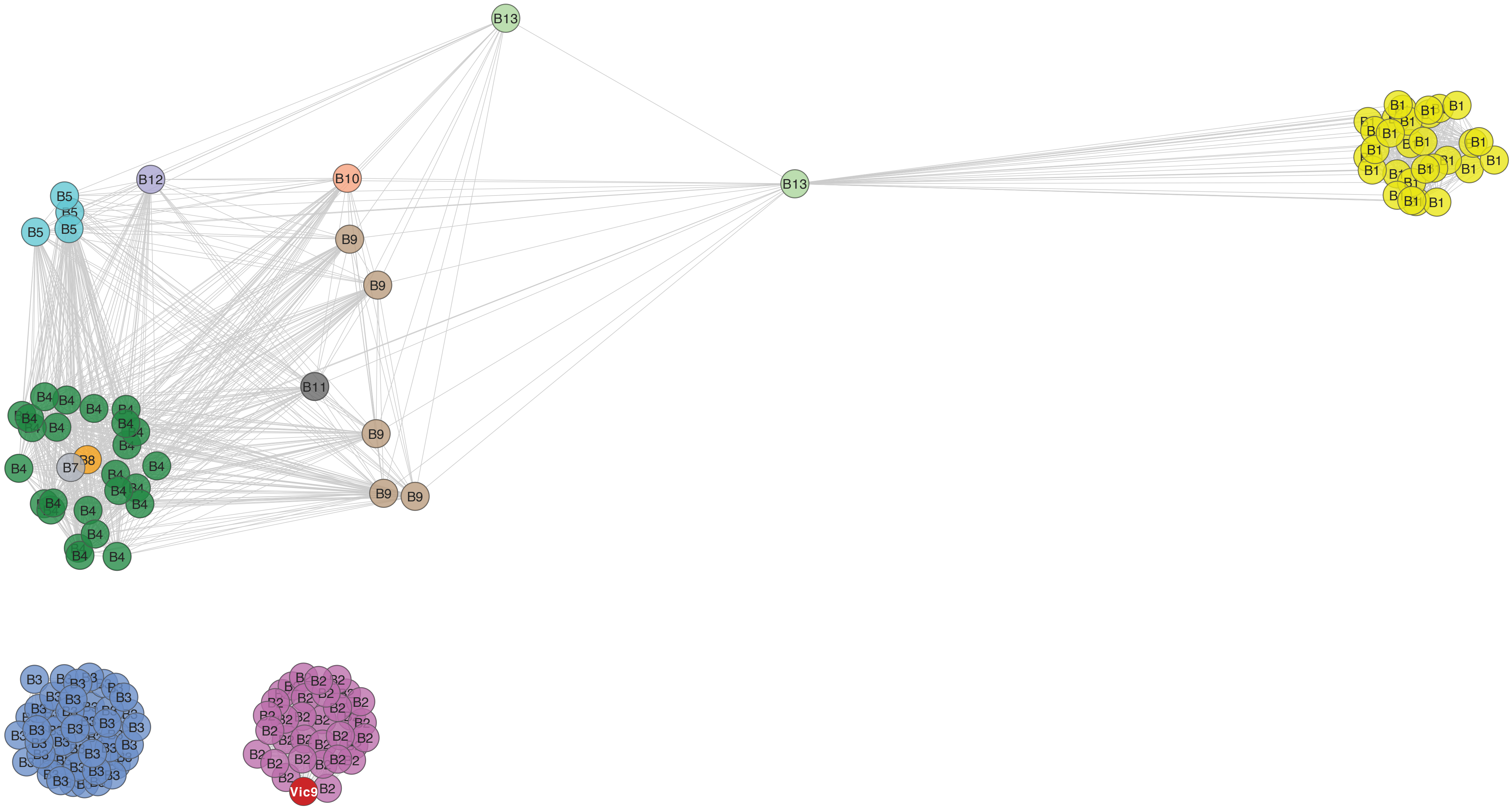

Supplementary Figure 2. Clustering of cluster B genomes based on gene similarity at the protein level.
Each node represents a cluster B mycobacteriophage sequence, with node color indicating different subclusters.
Genomes with significantly similar sequences are connected. The Vic 9 node is highlighted in red. The network is visualized using the edge-weighted spring-embedded model.
